# Priority gaps and promising areas in maternal health research in low- and middle-income countries: summary findings of a mapping of 2292 publications between 2000 and 2012

**DOI:** 10.1186/s12992-016-0227-z

**Published:** 2017-02-02

**Authors:** Matthew F Chersich, Greg Martin

**Affiliations:** 1 Wits Reproductive Health and HIV Institute, Faculty of Health Sciences, University of Witwatersrand, Johannesburg, South Africa; 2 Centre for Health Policy and MRC Health Policy Research Group, Faculty of Health Sciences, University of Witwatersrand, Johannesburg, South Africa; 3 Health Services Executive, Dublin, Ireland

**Keywords:** Maternal health, Health systems, Mapping study, Low and middle income country

## Abstract

This commentary sums the findings of a series of papers on a study that mapped the global research agenda for maternal health. The mapping reviewed published interventional research across low— and middle-income countries (LMICs) from 2000 to 2012, specifically focusing on investigating the topics covered by this research, the methodologies applied, the funding landscape and trends in authorship attribution.

The overarching aim underpinning the mapping activities was to evaluate whether research and funding align with causes of maternal mortality, and thereby highlight gaps in research priorities and governance. Fifteen reviewers from 8 countries screened 35,078 titles and abstracts, and extracted data from 2292 full-text articles.

Over the period reviewed, the volume of publications rose several-fold, especially from 2004 to 2007. The methodologies broadened, increasingly encompassing qualitative research and systematic review. Malaria and HIV research dominated over other topics, while sexually-transmitted infection research progressively diminished. Health systems and health promotion research increased rapidly, but were less frequently evaluated in trials or published in high-impact journals. Relative to disease burden, hypertension had double the publications of haemorrhage. Many Latin American countries, China and Russia had relatively few papers per billion US dollars Gross Domestic Product. Total LMIC lead authorships rose substantially, but only a quarter of countries had a local first author lead on >75% of their research, with levels lowest in sub-Saharan Africa. The median Impact Factor of high-income country led papers was 3.1 and LMIC-led 1.8. The NIH, USAID and Gates Foundation constituted 40% of funder acknowledgements, and addressed similar topics and countries.

The commentary notes that increases in outputs and broadening of methodologies suggest research capacity has expanded considerably, allowing for more nuanced, systems-based and context-specific studies. However, funders seemingly duplicate efforts, with topics and countries either receiving excessive or little attention. Better coordinated funding might reduce duplication and allow researchers to develop highly-specialised expertise. Repeated scrutiny of research agendas and funding may foment shifts in priorities. Building leadership capacity in LMICs and reconsidering authorship guidelines is needed.

## Background to mapping and main findings

Progress in achieving maternal health goals and reducing maternal deaths from individual conditions varies considerably across low— and middle-income countries (LMICs). Overall, considerably smaller reductions have occurred in deaths from haemorrhage and hypertension, than from HIV, for example. In principle, the research agenda in each country and region should closely reflect variations in outcomes and tackle the factors responsible for local inequities. Setting such an agenda and marshalling the resources to implement it does, however, require strong capacity at country level, and an alignment between a country’s priorities and the interests of foreign researchers and donors.

The MASCOT/MHSAR study (Multilateral Association for Studying health inequalities and enhancing North–south and South-South Cooperation; Maternal Health and Health Systems in South Africa and Rwanda) sought to build a clearer picture of this global research agenda by mapping interventional research in maternal health across LMICs from 2000 to 2012. It summed the topics covered, research methodologies applied, research funding landscape and the trends in authorship attribution between LMIC and high-income country (HIC) researchers. The study contrasted patterns in health systems and health promotion research with those in research on clinical conditions, specifically haemorrhage, hypertension, malaria, HIV and other sexually transmitted infections (STIs).

To our knowledge, this is the largest mapping of maternal health research, in terms of size, breadth of scope and extent of international collaboration. The mapping team involved 15 reviewers, drawn from 8 countries across 5 continents. Together they screened 35,078 titles and abstracts independently in duplicate, then assessed 4175 full text articles for eligibility, and extracted data from 2292 papers on 17 variables. The LMIC study was complemented by a mapping of literature on community-based interventions on maternal health in HICs, which located 119 studies on the topic. This commentary focuses on the LMIC study, but draws a few comparisons between the findings of the LMIC and HIC mappings. The study procedures in both mappings, similar to a ‘scoping review’, did not involve an evaluation of the quality of the included studies, or the extraction of data on the outcomes of interventions. It thus *synthesizes a body of literature*, as opposed to an *evaluation of literature*, as in a systematic review.

In this commentary, we sum the findings of the series of articles on the LMIC mapping and some implications thereof. Over the twelve years reviewed, the total number of publications in this field rose several-fold, with the largest rise occurring from 2004 to 2007. The range of research methods also broadened considerably, and increasingly encompassed the application of qualitative research and systematic review. Overall, articles on malaria and HIV dominated the field, with hypertension, haemorrhage and STI research making up less than a third of all papers on the clinical conditions. Most especially, in several parts of sub-Saharan Africa, a very high proportion of research addressed HIV. For example, in Cote d’Ivoire, almost 95% of studies in this period addressed HIV. Similarly, only 1 in 30 publications in South Africa was on haemorrhage in pregnancy. HIV research was two-fold more likely to apply qualitative methods than other clinical conditions, but, as compared to haemorrhage research, it was half as likely to include a health systems component in the intervention being studied. The number of publications per 1000 maternal deaths from hypertension was almost double that of haemorrhage.

Scant attention was placed on research about equity in the LMIC review. By contrast, the mapping of health promotion in HICs found that a third of studies targeted vulnerable groups, such as poor women, adolescents, and black and ethnic minorities. The timing of interventions studied also varied between LMICs and HICs. Two thirds of the HIC studies focused on the postpartum period, mostly addressing breastfeeding assistance and promotion, preventing and treating post-natal depression, or interventions to support and build capacity around parenting and child care. By contrast, 70% of LMIC studies addressed issues relating to women during pregnancy and much fewer included postpartum women.

Health system and health promotion articles increased over the review period at a faster rate than those on other topics, but were less likely to be evaluated in a trial, summarised in a systematic review, or published in high-impact journals. Studies in South Asia had a substantially greater focus on health systems or health promotion research than other regions. Research on STIs other than HIV, however, progressively diminished over time. The number of studies on STIs, malaria, hypertension and haemorrhage were very similar in 2000–2003, but diverged markedly thereafter. Merely 3.5% of studies addressed STIs in 2008–2012, and many of these were reviews, rather than empirical research.

In about ten countries, maternal health interventional research made up more than five percent of all health publications in the country. By contrast, in nearly twenty other countries, studies on maternal health contributed less than one percent to the overall research done in the country. In India, of note, maternal health research formed only 0.25% of all health publications from the country and there were only 0.13 publications per billion USD GDP. Many countries in Latin America and several large countries like China and Russia also had very few papers per billion USD GDP.

In terms of funding sources, the National Institute of Health, the US Agency for International Development, and the Bill and Melinda Gates Foundation together made up 40% of funder acknowledgements. For all types of funders, but especially for Global Health Initiatives, the most common topic studied was HIV and the most common region supported was sub-Saharan Africa. Few studies were funded by national governments, though this has slightly risen over time. Importantly, the two main causes of maternal mortality — haemorrhage and hypertension — had a high proportion of papers without any funder mention, as did STI articles. This suggests that researchers have continued their work in these critical areas, even in the absence of adequate support from funders. Funding patterns are quite different within HICs, where national governments were the largest funding source in the papers reviewed, followed by not-for-profit organisations.

The number of LMIC lead authorships, used as a measure of research capacity, rose substantially in the mid-2000s, but slowed thereafter. Findings on data ownership and a country’s ability to advance its own research agenda (as measured as the proportion of articles led by authors affiliated with an LMIC institution) were concerning. Only about half the papers across the review period were led by an LMIC author, with a further 8% having a dual HIC and LMIC affiliation. And, only a quarter of countries led more than 75% of their research, while another quarter led less than 25%. Very few countries increased the proportion of articles led over time; in some it even decreased.

Authors affiliated with institutions in the United States and United Kingdom together accounted for a third of all publications. Authors from these two countries were four times less likely than those from institutions in mainland Europe to also hold an affiliation in a LMIC. Of note, few authors held affiliations in more than one LMIC. Two thirds of studies funded by USAID and the European Union were led by HIC researchers, twice as many as that of Wellcome Trust and Rockefeller Foundation. Even in studies acknowledging funding by national governments in LMICs, only about 70% had an LMIC lead. Strikingly, the median Impact Factor was 3 · 1 for papers led by a HIC author and 1 · 8 for those from LMICs. In fact, HIC researchers lead two thirds of articles in journals with an Impact Factor above 5, and even higher proportions of systematic review and modelling studies. Sub-Saharan Africa had the lowest overall proportion of locally-led publications of all geographical regions.

## Conclusions and implications of the findings

The large rise in total research outputs and broadening of the range of methods employed over the twelve year period reviewed indicates a major expansion in the number of researchers studying maternal health and in the diversity of their skill sets. The apparent maturation of maternal health research in LMICs, accompanied by an expansion of subtlety and scope, potentially allows for an increasingly nuanced approach to addressing research questions. The rapid rise in health systems, health promotion and qualitative research also likely reflects a cognisance that context, social dimensions and systems determine the effectiveness of health interventions. These factors, if aligned with information needs and funding, bode well for the future of maternal health research. A number of concerns in the current state of maternal health research remain, however. Below we detail some of these concerns and suggest ways in which they might be addressed.

The mapping findings strongly call for higher funding levels for maternal health in general, and that such funding better reflects the distribution of maternal ill health and the causes thereof. Allocations for a number of maternal health conditions are dwarfed by funding for HIV research. Additional resources are especially needed for research on haemorrhage and hypertension in sub-Saharan Africa; but also for South Asian countries and for other STIs worldwide. The neglect of STI research is especially concerning given the considerable burden of these infections and the global efforts to eliminate mother-to-child transmission of syphilis. Maternal health is accorded high priority by global and national policy makers and donors, a fact that portends well for efforts to garner additional funding. Translating these sentiments into funding, however, will require measures to convince governments, philanthropic agencies and other donors of what could be achieved, and incentives for pharmaceutical investment.

Currently, a few major funders dominate the field, and they address similar topics and geographical areas. Almost certainly this results in duplication of efforts, and diffuses the focus of funders and researchers, who develop a broad knowledge of several topics, rather than an in-depth understanding of a few. Clearly it is necessary to have some researchers with a broad understanding of a research field, but equally there are benefits to having experts with more focused specialised expertise. Moreover, particular topics and countries then either receive concerted attention from the large funders, or get little or no attention at all. Conditions that are accorded low priority, such as STIs, have few alternative funding sources, and the progression of knowledge on these topics stagnates.

An alternative approach is worth considering. This would involve a coordinated effort to develop a long-term, carefully planned, progressive accumulation of knowledge on a topic, produced by teams of funders and researchers who steadily acquire the specialised skills required for systematically advancing a topic. Key funders would then each take responsibility for research on certain topics and regions, and develop specialised expertise both within their organisation and in research teams. This level of coordination would necessitate sharing of information between funders, and joint priority setting, planning and action. Practically, coordination would encompass the prioritisation of maternal health research topics, redirection of research funding and the systematic addressing of research capacity gaps. The finding that few authors in LMICs hold affiliations in another LMIC suggests that interaction and thus coordination between researchers in different LMICs is weak. Indeed, an agenda driven by researchers across LMICs seems a far-.off goal in the current context where HIC researchers appear to dominate the terms of collaboration. A Global Health Initiative for maternal health, similar to those for HIV and malaria for example, could go a long way towards coordinating the research agenda and re-directing funding. It might overcome market failures in technologies and drugs for maternal health, as has been done for conditions such as HIV and malaria. To be successful, however, this would have to fairly represent the interests of all those involved in this field.

Stronger research leadership in LMICs might go a long way towards addressing many of the concerns raised here. The mapping identified that the high levels of HIC authorship have persisted over time, signalling deficits in local ownership of data, as well as in internal capacity to analyse and articulate research findings. To redress this, research funding might include more mentorship and specific funded opportunities for developing research ideas, analysing data and completing publications. Too often, money is only provided to LMIC researchers for the technical tasks of research, such as running research sites, and recruiting and retaining study participants. These tasks consume the energies of local researchers, and are not accorded value in authorship guidelines. The dominance of HIC authors on studies supported by some funders might indicate important variations in expectations of authorship, and discrepancies in how researchers and funders understand the concept of partnership. International guidelines on authorship are seemingly not applied uniformly. Journals themselves also have a role to play in placing LMIC and HIC researchers on a more equal footing. As part of article processing fees, journals could, for example, provide more statistical and editing assistance and give constructive feedback on ways to improve these aspects of a paper prior to peer review. It also seems that journals accord less value to health systems research, which, even if of good quality, is less likely to be published in high-impact journals than clinical studies.

Gaps in systematic review expertise in LMICs are especially concerning, given the importance of these skills for evaluating and synthesizing locally-relevant evidence to inform policy decisions. Also, the near absence of health systems, health promotion and qualitative research on some conditions might suggest that research on these conditions still tends to adopt an over-medicalised approach. It is important, however, to note that identifying a research gap does not necessarily signal a dissonance between the knowledge needs and the research done. Knowledge needs vary between fields, and differences in number of studies and in their design might be appropriate. Implementation scale-up, supported by health systems evidence, for example, might be the present priority in a field. This perhaps partly explains why many of the studies on haemorrhage included a health systems component and interventions were evaluated in a trial.

Tracking whether the burden of different conditions matches the research funding and outputs in different settings, and the level of inter-funder coordination, might promote accountability and advances in research governance. Research governance also includes documenting the influence of funders and researchers from HICs on research processes and authorship attribution. Standardising the reporting of funder roles, the types of funding provided and the support given for local research capacity and authorship, would promote transparency about funders’ contributions, improve understanding of how funders and researchers interact, and incentivise improvements in these areas. In future, much of this monitoring could be done using text mining techniques, considerably reducing the effort and time taken by mapping studies such as this one.

Lastly, it is worth noting that funding and research priorities are likely to shift in the era of the Sustainable Development Goals. These changes will potentially strengthen proponents of research that specifically addresses gaps in health outcomes between populations and between conditions. Potentially, the representation of the major causes of maternal mortality, such as haemorrhage, could rise substantially. Taking advantage of opportunities in this new era will, however, require a carefully crafted research agenda reflecting knowledge needs and proactive steps to shift research funding. Ongoing scrutiny of research funding distribution and of donor coordination is warranted, alongside efforts to address the specific capacity and leadership constraints in different settings. Specifically, more stringent authorship oversight and reconsideration of authorship guidelines could accelerate the growth in LMIC leadership and counter some of the inequities between LMIC researchers and their more prominent HIC counterparts. Against the background of a strategic repositioning of maternal health within the Sustainable Development Goals, such measures to re-align the global research agenda might just usher in a brighter era for maternal health.

## Box 1: Summary recommendations drawn from findings of the MASCOT MH-SAR mapping

Strategically reposition maternal health within the Sustainable Development Goals, especially concerning the potential for research on health systems, health promotion and conditions such as haemorrhage to reduce the differential health outcomes between population groups.

Advocate for funding to better reflect the distribution of maternal ill health and the causes thereof, with more attention given to haemorrhage and hypertension research in sub-Saharan Africa, to research in South Asian countries in general and to studies on STIs worldwide.

Establish a Global Health Initiative for maternal health, similar to those for HIV and malaria, which coordinates the research agenda and funding thereof.

Translate the high priority given to maternal health by policy makers and donors into increased research funding, by convincing them of what could be achieved with increased resources.

Apply the considerably increased extent and diversity of research capacity in LMICs to address locally-relevant research questions and to extend the gains made in health systems and health promotion research.

Optimise funder coordination, with information sharing, joint priority setting, redirection of research resources and each funder taking responsibility for advancing knowledge on certain topics and regions so as to avoid duplication of efforts.

Develop a long-term coordinated research strategy that allows for teams of researchers and funders to accrue specialised skills and systematically advance knowledge on a topic.

Strengthen research leadership in LMICs by, for example, including more funded opportunities for developing research ideas, analysing data and completing publications.

Include considerations specific to LMIC-HIC research partnerships within international authorship guidelines, such as according value to the technical tasks of research in LMICs (running research sites, and recruiting and retaining participants).

As part of research governance, track authorship practices of different funders and HIC research institutions, aiming to understand reasons for discrepancies in authorship attribution, and how these might be rectified.

Journals play a larger role in building research leadership in LMICs, by, for example, using part of article processing fees to provide editing and statistical assistance, and advice on improving articles prior to peer review.

Within declaration made on journal articles about research funders, require funders to report on their support given for local research capacity and authorship within the study concerned, if any.

Ongoing monitoring, based around text mining techniques to cover: the research topics addressed and whether these match the burden of maternal health conditions; research methodologies applied; research funding and outputs in different settings; and the influence of funders and researchers from HICs on authorship attribution authorship patterns between settings and funders.

## Abbreviations

HICs: High-income countries
LMICs: Low— and middle-income countries
MASCOT: Multilateral association for studying health inequalities and enhancing north–south and south-south COoperaTion
MH-SAR: Maternal health and health systems in South Africa and Rwanda
STIs: Sexually transmitted infections

